# Prevalence and Influencing Factors of Central Obesity among Adults in China: China Nutrition and Health Surveillance (2015–2017)

**DOI:** 10.3390/nu16162623

**Published:** 2024-08-09

**Authors:** Jing Nan, Mulei Chen, Hongtao Yuan, Shuya Cai, Wei Piao, Fusheng Li, Yuxiang Yang, Liyun Zhao, Dongmei Yu

**Affiliations:** 1 National Institute for Nutrition and Health, Chinese Center for Disease Control and Prevention, Beijing 100050, China; nj13939012762@163.com (J.N.); yuanht@ninh.chinacdc.cn (H.Y.); caisy@ninh.chinacdc.cn (S.C.); piaowei@ninh.chinacdc.cn (W.P.); 18246668833@163.com (F.L.); yangyuxiang1996@sina.com (Y.Y.); zhaoly@ninh.chinacdc.cn (L.Z.); 2 Chinese Center for Disease Control and Prevention, Beijing 102206, China; chenml@chinacdc.cn; 3 NHC Key Laboratory of Public Nutrition and Health, Chinese Center for Disease Control and Prevention, Beijing 100050, China

**Keywords:** central obesity, adults, influencing factors, China

## Abstract

The purpose of this study was to describe the prevalence of central obesity and its influencing factors among Chinese adults aged 18 or older. The data were from China Nutrition and Health Surveillance (2015–2017), which used a stratified, multistage, random sampling method. A total of 145,298 adults aged 18 years or older from 31 provinces were included in this study. The Criteria of Weight for Adults promulgated by China in 2013 were used to determine central obesity. Out of all the adults investigated, 48,342 were identified with central obesity, with a prevalence rate of 33.3%. A logistic analysis suggested that the following factors were associated with central obesity: female sex [odds ratio (OR) = 1.329, 95%CI = 1.277~1.384]; increasing age [OR (95%CI): 1.146 (1.061~1.238), 1.254 (1.167~1.348), 1.774 (1.651~1.907), 2.041 (1.894~2.198), 2.434 (2.239~2.647)]; being married [OR = 1.184, 95%CI = 1.077~1.302]; being divorced or widowed [OR = 1.132, 95%CI = 1.006~1.273]; an urban setting [OR = 1.096, 95%CI = 1.061~1.132]; BMI [OR (95%CI): 0.159 (0.095~0.266), 12.645 (11.388~14.042), 180.989 (153.025~214.064)]; drinking [OR = 1.069, 95%CI = 1.031~1.109]; and screen time > 5 h [OR = 1.088, 95%CI = 1.036~1.141] were risk factors for central obesity; while education above primary school [OR (95%CI): 0.905 (0.875~0.936), 0.857 (0.802~0.915)] and sufficient physical activity [OR = 0.819, 95%CI = 0.782~0.858] were protective factors for central obesity. This study revealed that the prevalence of central obesity, which differed by gender and age, is still high. Large differences between different groups and geographic regions exist persistently. Effective, sustainable, and culturally targeted interventions are needed.

## 1. Introduction

With the continuous development of the social economy and the intensification of the aging process, people’s lifestyles are changing; the occurrence of obesity and its health risks are increasing, which has become a major global health challenge [1]. Obesity is classified into general obesity and central obesity based on the different positions of adipose tissue accumulation in the body [2]. The condition characterized by excessive fat accumulation in the abdomen is referred to as central obesity. Relevant studies have demonstrated that central obesity exhibits a stronger association with numerous chronic diseases compared to general obesity, thereby increasing the risk of cardiovascular and cerebrovascular diseases, hypertension, type 2 diabetes, and metabolic syndrome [3]. Moreover, there is a high incidence of central obesity in both developed and developing countries, and central obesity has become an important public health problem in our country, China, in recent years [4].

To gain timely insights into the prevalence and influencing factors of central obesity among Chinese adults, as well as provide a reference for prevention and control, this study analyzed nationwide data from China Nutrition and Health Surveillance (2015–2017) and described the prevalence and influencing factors associated with central obesity among Chinese adults aged 18 years old or above.

## 2. Materials and Methods

### 2.1. Data Source

The data were from China Nutrition and Health Surveillance (2015–2017). This was a survey among adults in 2015, in which a stratified, multistage, random sampling method was utilized to recruit representative participants from 31 provinces/municipalities/autonomous regions in mainland China. Detailed information about this survey can be found in our previous report [5]. The study protocol for the survey year of 2015 was approved by the Ethics Committee of the National Institute for Nutrition and Health, as well as the Chinese Center for Disease Control and Prevention (approval number: 201519-B). Before participating in the study, all individuals provided informed consent. A total of 145,298 sample participants aged 18 years or older were included in the analysis.

### 2.2. Data Collection

China Nutrition and Health Surveillance (2015–2017) collected information through four parts: questionnaire survey, physical examination, dietary evaluation, and laboratory test. All surveys were conducted by trained local CDC staff using uniform questionnaires and the same brand and model of instruments. The data from the first two parts were mainly used in this study. Face-to-face questionnaires were used to collect information on the sample participants, including lifestyle factors, physical activity, and health status. The physical examination involved in this study included height, weight, and waist circumference. Height, weight, and waist circumference were measured by trained staff according to standard methods using a TZG height sitter (Suzhou Kangyang Automation Co., Ltd., Suzhou, China), a TANITA HD-390 electronic scale (TANITA (Shanghai, China) Trading Co., Ltd., Shanghai, China), and a waist measure ruler. These tools were all made in China and accurate to 0.1 cm, 0.1 kg, and 0.1 cm, respectively.

### 2.3. Definition of Central Obesity

In 2013, the Criteria of Weight for Adults promulgated by China proposed that a male waist circumference ≥90 cm and a female waist circumference ≥85 cm can be judged as central obesity. This study used this criterion to determine the central obesity of the sample participants.

### 2.4. Confounding Factors

A wide range of potential confounders were accounted for: (1) gender included male and female; (2) the age group consisted of six groups, namely 18 to 29 years old, 30 to 39 years old, 40 to 49 years old, 50 to 59 years old, 60 to 69 years old, and 70 years old and above; (3) education level was classified as low (primary school or below), moderate (junior school), and high (high school or above); (4) marital status included unmarried, married, and divorced/widowed; (5) residence included urban and rural areas; (6) region was divided into 7 groups according to location, namely Northern China, Northeast China, Eastern China, Central China, Southwest China, Northwest China, and Southern China; (7) according to the average annual household income quartile, income was classified as low (<CNY 5000), moderate (CNY 5000~9999), high (CNY 10,000~18,999), and very high (≥19,000); (8) body mass index (BMI) was classified as low(<18.5 kg/m^2^), normal (18.5≤ to <24 kg/m^2^), overweight (24≤ to <28 kg/m^2^), and obese (≥28 kg/m^2^); (9) smoking status was classified as never smoked, formerly smoked, or currently smoke; (10) alcohol consumption was classified as no current consumption (no consumption in the last 30 days or never consumed) and current consumption (had consumption in the last 30 days); (11) physical activity was defined as insufficient if the total time of moderate-intensity activity was less than 150 min within one week, high-intensity activity was less than 75 min, or the cumulative amount of moderate- and high-intensity activity was less than 150 min and otherwise it was defined as sufficient activity; (12) sleep duration was categorized as ≤6 h, 7~8 h, and ≥9 h per day; finally, (13) excessive screen time was defined as more than 5 h per day and normal screen time was defined as less than 5 h per day or 5 h per day.

### 2.5. Quality Control

To ensure the quality of the data, the National Project Working Group developed a quality control plan and supervised its implementation. The initiative ensured the implementation of research with uniform programs, manuals, questionnaires, training and assessments, equipment, and reagents. In addition, researchers carried out unified data entry and data cleaning for each survey site to ensure data quality.

### 2.6. Statistical Analysis

SAS 9.4 software (SAS Institute Inc., Cary, NC, USA) was used to analyze the data. When describing the general characteristics of the sample participants, variables were expressed as N (%). The Rao–Scott chi-square test based on sampling design adjustment was used to compare the central obesity rate among disordered classification groups, and the chi-square trend test was used for the trend in rate change with age group. The PROC SURVEYFREQ was used to calculate the standardized prevalence and 95%CI of central obesity. The weight was derived from the standard population published by the China National Bureau of Statistics in 2010. The influencing factors of central obesity were analyzed using a multivariate unconditional logistic regression model. A two-sided *p*-value < 0.05 was defined as statistical significance.

## 3. Results

### 3.1. General Characteristics of the Participants

A total of 145,298 sample participants were included, of whom 67,535 were men (46.5%) and 77,763 were women (53.5%). The mean age of the female adults in this study was higher than that of the male adults. In terms of residence, 61,155 (42.1%) were urban residents and 84,143 (57.9%) were rural residents. Of the sample participants, 10,139 (7.0%) were current smokers, 39,920 (27.5%) were alcohol drinkers, and 129,299 (89.0%) had sufficient physical activity. In addition, the average BMI, average sleep time, and average screen time were (24.13 ± 0.07) kg/m^2^, (7.75 ± 0.02) hours, and (3.58 ± 0.07) hours, respectively (Table 1).

### 3.2. Central Obesity among Adults with Different Characteristics

The standardized prevalence of central obesity among Chinese adults aged 18 years or older was 29.9%, with a significantly higher prevalence in men than in women (31.0% vs. 28.8%). Among the different age groups, the highest prevalence was found in the age group of 60–69 years (38.0%) and the lowest prevalence was found in the age group of 18–30 years (18.4%). In terms of socio-economic factors, the prevalence of Northern China residents (38.9%) was higher than that of other areas; the prevalence of divorced or widowed people (33.9%) was higher than that of unmarried people, and that of the group with a higher income level (31.3%) was higher than that of the group with a lower income level. The prevalence decreased with the increase in educational level and increased with the increase in body mass index (*p* < 0.001). In addition, people who smoke or drink alcohol have a higher prevalence of central obesity than the general population, while people who have less screen time have a lower prevalence than the general population (*p* < 0.001). The shorter the sleep time, the higher the prevalence, and the highest prevalence was in the ≤6 h group (33.9%).

Of all the adults investigated, 48,342 were identified with central obesity. There were significant differences in central obesity prevalence by gender, age, education level, marital status, region of the country, BMI, smoking status, alcohol consumption, sleep duration, and screen time (*p* < 0.05, Table 2).

### 3.3. Influencing Factors of Central Obesity Prevalence

To obtain the influencing factors of central obesity in Chinese adults, this study conducted a logistic regression analysis. In this study, the dependent variable is whether Chinese adults suffer from central obesity, and the independent variables were gender, age, education level, marital status, residence, region, average annual household income, BMI, smoking status, drinking status, physical activity, sleep duration, and screen time. After controlling for other factors, the results showed that the risk of central obesity in women was higher than that in men (OR = 1.329, 95%CI = 1.277~1.384). Taking the 18-year-old group as the reference, the risk of central obesity increased with age, and the risk of central obesity was the highest in the ≥70-year-old group (OR = 2.434, 95%CI = 2.239~2.647). The following factors were associated with a higher risk of central obesity: living in an urban setting (OR = 1.096, 95%CI = 1.061~1.132); living in Northern China [OR (95%CI): 0.644 (0.608~0.682), 0.752 (0.718~0.787), 0.732 (0.694~0.772), 0.825 (0.781~0.871), 0.777 (0.737~0.820), and 0.579 (0.541~0.618) for Northeast, Eastern, Central, Southwest, Northwest, and Southern China, respectively]; being married (OR = 1.184, 95%CI = 1.077~1.302); having a higher average annual household income [OR (95%CI): 1.047 (1.004~1.093), 1.067 (1.019~1.118) for high level and very high level, respectively]; being overweight and obese [OR (95%CI): 12.645 (11.388~14.042), 180.989 (153.025~214.064) for overweight and obese, respectively]; being a current smoker (OR = 1.117, 95%CI = 1.050~1.188); being a current consumer of alcohol (OR = 1.069, 95%CI = 1.031~1.109); engaging in screen time longer than 5 h (OR = 1.088, 95%CI = 1.036~1.141). Middle school education and above [OR (95%CI): 0.905 (0.875~0.936), 0.857 (0.802~0.915) for moderate and high education levels, respectively] and sufficient physical activity (OR = 0.819, 95%CI = 0.782~0.858) were protective factors associated with central obesity (Table 3).

## 4. Discussion

With economic development and changes in dietary patterns, obesity, especially central obesity, has become an important public health problem in China. Compared with general obesity, central obesity is more closely related to the increase in the prevalence of cardiovascular disease, cancer, and other chronic diseases. Therefore, it is necessary to study the current situation and the factors that influence adult central obesity in China. Based on the data of the China Nutrition and Health Surveillance (2015–2017), this study analyzed the prevalence of central obesity among adults with different characteristics in China and explored the potential influencing factors. We found that the standardized prevalence of central obesity was 29.9%. The prevalence of males was significantly higher than that of females (31.0% vs. 28.8%, *p* < 0.001). And there was a significantly higher prevalence in those living in Northern China than in Southern China (38.9% vs. 20.0%, *p* < 0.001). Age, education levels, marital status, income level, BMI, smoking, drinking, physical activity, and screen time were all influencing factors related to central obesity, and the controllable factors should be addressed as the key factors for the prevention of central obesity.

Understanding the prevalence of central obesity in Chinese adults is helpful to further understand the health status of Chinese people. Compared with the monitoring data in 2012, the rate of central obesity has increased, which is consistent with the results of Ma’s related research [6]. This study shows that the prevalence of central obesity in men is higher than that in women. Similar to Zhang’s research results [7], Zhang studied the prevalence of central obesity among adults aged 18–35 years in 15 provinces in China from 1993 to 2018. The results showed that the prevalence of central obesity in men increased from 4.40% to 35.49%, and the prevalence of central obesity in women increased from 6.33% to 18.31%, with an average annual growth rate of 8.14% and 2.58%, respectively. The higher prevalence of central obesity in men than women may be related to the fact that women pay more attention to their external contours and are more willing to control their weight than men [8]. We also found the rate of central obesity in urban and rural areas gradually converging, which may be related to the fact that the increase in central obesity in rural areas is much greater than that in urban areas, mentioned in the study of NCD Risk Factor Collaboration (NCD-RisC) [9]. This result suggests that rural central obesity needs to be highly valued, and prevention and control should be strengthened.

Understanding which factors are associated with central obesity can help identify individuals at greater risk of central obesity. The results of this study showed that the influencing factors associated with central obesity can be divided into socio-economic factors and lifestyle factors. In terms of individual socio-economic factors, gender, age, education level, marital status, residence, region, income, and BMI were all related to central obesity [10]. Women were more likely to suffer from central obesity than men. Previous studies from different settings in the United States, India, Iran, and West Africa have also endorsed this funding [11,12,13,14], which may be related to women’s greater emphasis on diet and their external attention. It is worth mentioning that although women have a higher risk of disease, the central obesity prevalence of women in this study is slightly lower than that of men, which may be related to women’s genetic changes, hormone metabolism, and the need to assume reproductive responsibilities [10,15]. The prevalence of central obesity tended to increase with increasing age, which was consistent with Hu’s result [16]. This result may be related to the gradual decline in physical activity and activity levels in adults as they age. In terms of education level, people with high education levels have a lower risk of central obesity than those with low education levels, which is similar to the findings from Iran [13]. This may be because, with the improvement of education level, adults would like to pay more attention to healthy eating and exercise, and when receiving health education, they are more likely to take the initiative to transform it into a practice that is beneficial to themselves [17]. As for marital status, married, divorced, or widowed people have a higher risk of central obesity than unmarried people, which may be related to people’s emotional mentality and hormonal readiness [18]. The conclusions of different countries are not the same, and further research is needed to verify this view. People living in urban areas had a higher risk of disease than those living in rural areas, which was consistent with India’s conclusion [12]. The frequency of eating out and high-fat and high-salt diets is higher than that of rural residents, and urban workers have longer sedentary time, less physical activity, and greater stress, which may lead to overeating and central obesity [19]. Moreover, the risk of central obesity in Northern China is higher, which may be caused by population distribution, economic development level, and environmental factors [20]. This result was the same as most of the research results of Chinese scholars and also conformed to the regional distribution of China’s economic development [7]. In terms of family annual per capita income, the risk of central obesity in high-income groups was higher than that in low-income groups, which may be related to the higher number of out-of-home meals and the higher level of living and entertainment in high-income groups [21]. This result was consistent with Neha Shri’s study [12], but unlike Wang Ru’s study [22], it may be due to the different sample sizes and exclusion criteria of these studies. In this study, there were still cases of central obesity in groups with normal BMI or even low BMI, which suggested that central obesity determined by waist circumference was more sensitive than obesity determined by BMI, and the growth rate of adult central obesity rate may exceed the obesity rate determined by BMI [23,24].

In terms of the influencing factors of lifestyle, drinking, physical activity, and screen time all had an influence on central obesity. Bosomworth’s research results also support this conclusion [25]. The risk of central obesity was higher in the drinking group, which was consistent with the results of S Lourenço’s research. This may be due to the high calories of alcohol, which is a non-negligible source of carbon and water, and alcohol hinders fat burning and increases hunger. People with drinking habits were often accompanied by excessive energy intake [26]. In addition, people with sufficient physical activity or short screen times had a lower risk of central obesity than the general population, which was the same as the results of Daniel Kim’s related research on American residents [27] and was in line with the recommended trend of the Chinese Dietary Guidelines (2022) [28]. This may be because the screen behavior was always accompanied by sedentary and insufficient physical activity, and the risk of central obesity in people with sufficient physical activity was lower than that in people with insufficient physical activity [29].

The data of this study are representative nationwide. After weighted adjustments, this study better reflects the epidemiological characteristics of central obesity among adults aged 18 or over in China. However, this study also has some limitations. Firstly, it was a cross-sectional study, so the causal relationship cannot be determined. Secondly, subjects may have a memory bias when conducting the survey because participants self-reported their demographics, behavior, and certain health factors. Thirdly, although this study adjusted for a wide range of confounding factors, it did not take into account remaining confounding factors, such as dietary factors, shift work, psychological stress, and so on [30,31,32].

## 5. Conclusions

This study provides a comprehensive description of the central obesity epidemic in China, related disparities, and influencing factors across population groups based on recent national survey data. The results showed that women, high BMI, age, smoking, alcohol consumption, and a lack of physical activity were the main risk factors for central obesity, and increased educational level and sufficient physical activity were protective factors against central obesity. Intervention for controllable factors can reduce adult waist circumference and central obesity rates.

## Figures and Tables

**Table 1:** Characteristics of participants stratified by sex.

	Men (*N*, %) *	Women (*N*, %) *	Total (*N*, %) *
*N*	67,535 (46.5)	77,763 (53.5)	145,298 (100)
Age			
18~29	5713 (3.9)	7048 (4.9)	12,761 (8.8)
30~39	7867 (5.4)	9960 (6.9)	17,827 (12.3)
40~49	14,791 (10.2)	18,163 (12.5)	32,954 (22.7)
50~59	16,494 (11.4)	19,387 (13.3)	35,881 (24.7)
60~69	15,303 (10.5)	16,315 (11.2)	31,618 (21.7)
≥70	7367 (5.1)	6890 (4.7)	14,257 (9.8)
Education level			
Low	26,502 (18.2)	41,776 (28.8)	68,278 (47.0)
Moderate	35,406 (24.4)	30,103 (20.7)	65,509 (45.1)
High	5627 (3.9)	5884 (4.0)	11,511 (7.9)
Marital status			
Unmarried	3753 (2.6)	2212 (1.5)	5965 (4.1)
Married	61,725 (42.5)	70,757 (48.7)	132,482 (91.2)
Divorced/Widowed	2057 (1.4)	4794 (3.3)	6851 (4.7)
Residence			
Urban	27,551 (19.0)	33,604 (23.1)	61,155 (42.1)
Rural	39,984 (27.5)	44,159 (30.4)	84,143 (57.9)
Region of China			
Northern	9949 (6.8)	11,579 (8.0)	21,528 (14.8)
Northeast	6913 (4.8)	7870 (5.4)	14,783 (10.2)
Eastern	18,748 (12.9)	20,944 (14.4)	39,692 (27.3)
Central	8330 (5.7)	9906 (6.8)	18,236 (12.6)
Southwest	8972 (6.2)	11,021 (7.6)	19,993 (13.8)
Northwest	9205 (6.3)	9992 (6.9)	19,197 (13.2)
Southern	5418 (3.7)	6451 (4.5)	11,869 (8.2)
Average annual household income			
Low	15,747 (10.8)	17,611 (12.1)	33,358 (22.9)
Moderate	15,843 (10.9)	18,439 (12.7)	34,282 (23.6)
High	18,951 (13.0)	22,186 (15.3)	41,137 (28.3)
Very high	16,994 (11.7)	19,527 (13.4)	36,521 (25.1)
BMI			
Low	2246 (1.6)	2913 (2.0)	5159 (3.6)
Normal	31,202 (21.5)	35,859 (24.7)	67,061 (46.2)
Overweight	24,637 (17.0)	27,098 (18.6)	51,735 (35.6)
Obese	9450 (6.5)	11,893 (8.2)	21,343 (14.7)
Smoking			
Never	23,224 (16.0)	75,003 (51.6)	98,227 (67.6)
Former	34,814 (23.9)	2118 (1.5)	36,932 (25.4)
Current	9497 (6.5)	642 (0.4)	10,139 (6.9)
Alcohol consumption			
No current alcohol consumption	35,476 (24.4)	69,902 (48.1)	105,378 (72.5)
Current alcohol consumption	32,059 (22.1)	7861 (5.4)	39,920 (27.5)
Physical activity			
Insufficient	8430 (5.8)	7569 (5.2)	15,999 (11.0)
Sufficient	59,105 (40.7)	70,194 (48.3)	129,299 (89.0)
Sleep duration			
≤6 h	12,293 (8.5)	14,799 (10.2)	27,092 (18.7)
7~8 h	41,312 (28.4)	45,856 (31.6)	87,168 (60.0)
≥9 h	13,930 (9.6)	17,108 (11.8)	31,038 (21.4)
Screen time			
≤5 h	58,740 (40.4)	69,595 (47.9)	128,335 (88.3)
>5 h	8795 (6.1)	8168 (5.6)	16,963 (11.7)

* The data outside the brackets indicate the number of subjects, and the data inside the brackets indicate the composition ratio (%). Due to rounding, the value of multi-category variables may not reach 100%. Abbreviations: BMI, body mass index.

**Table 2:** Comparison of central obesity among adult residents with different characteristics in China.

	Prevalence (%)	95%CI	Rao–Scott X^2^	*p*-Value
Total	29.9	28.4~31.4		
Sex			10.15	0.0014
Men	31.0	29.2~32.8		
Women	28.8	27.3~30.3		
Age			545.33	<0.0001
18~29	18.4	16.6~20.2		
30~39	28.1	25.5~30.7		
40~49	33.5	32.0~35.0		
50~59	37.9	36.2~39.6		
60~69	38.0	36.1~39.9		
≥70	34.9	32.5~37.4		
Education level			32.19	<0.0001
Low	32.7	31.0~34.3		
Moderate	29.6	27.8~31.4		
High	25.0	22.4~27.7		
Marital status			222.80	<0.0001
Unmarried	16.2	13.7~18.6		
Married	31.7	30.3~33.2		
Divorced/Widowed	33.9	30.9~36.9		
Residence			1.81	0.1787
Urban	30.9	28.6~33.3		
Rural	28.8	26.9~30.7		
Region of China			77.80	<0.0001
Northern	38.9	36.1~41.6		
Northeast	33.0	29.9~36.0		
Eastern	30.8	28.1~33.5		
Central	28.9	25.0~32.8		
Southwest	25.2	23.1~27.2		
Northwest	30.0	26.9~33.1		
Southern	20.0	16.5~23.6		
Average annual household income			5.71	0.1264
Low	29.5	27.7~31.4		
Moderate	28.5	27.0~30.0		
High	31.3	29.5~33.0		
Very high	30.0	27.2~32.7		
BMI			397,694.71	<0.0001
Low	0.8	0.4~1.2		
Normal	5.3	4.6~6.0		
Overweight	42.3	40.8~43.8		
Obese	90.7	89.6~91.7		
Smoking			47.15	<0.0001
Never	29.5	27.9~31.0		
Former	29.5	27.7~31.3		
Current	37.8	35.3~40.3		
Alcohol consumption			15.86	<0.0001
No current consumption	29.1	27.7~30.5		
Current consumption	31.9	29.8~33.9		
Physical activity			3.23	0.0725
Insufficient	31.2	29.1~33.4		
Sufficient	29.7	28.3~31.2		
Sleep duration			41.70	<0.0001
≤6 h	33.9	32.1~35.7		
7~8 h	29.4	27.9~30.9		
≥9 h	28.7	26.6~30.7		
Screen time			37.46	<0.0001
≤5 h	30.8	29.2~32.3		
>5 h	26.4	24.6~28.2		

Abbreviations: BMI, body mass index.

**Table 3:** Logistic regression analysis results of influencing factors.

Influencing Factor		*β*	SE	Wald *X*^2^	*p*	OR	95%CI
Intercept		−5.018	0.139	1297.975	<0.0001		
Sex	Women vs. Men	0.285	0.021	192.565	<0.0001	1.329	1.277~1.384
Age	30~39 vs. 18~29	0.137	0.039	12.100	0.0005	1.146	1.061~1.238
	40~49 vs. 18~29	0.227	0.037	37.931	<0.0001	1.254	1.167~1.348
	50~59 vs. 18~29	0.574	0.037	244.079	<0.0001	1.774	1.651~1.907
	60~69 vs. 18~29	0.713	0.038	352.559	<0.0001	2.041	1.894~2.198
	≥70 vs. 18~29	0.890	0.043	435.930	<0.0001	2.434	2.239~2.647
Education level	Moderate vs. Low	−0.100	0.017	33.447	<0.0001	0.905	0.875~0.936
	High vs. Low	−0.155	0.034	21.205	<0.0001	0.857	0.802~0.915
Marital status	Married vs. Unmarried	0.169	0.049	12.149	0.0005	1.184	1.077~1.302
	Divorced/Widowed vs. Unmarried	0.124	0.060	4.271	0.0388	1.132	1.006~1.273
Residence	Urban vs. Rural	0.092	0.017	30.996	<0.0001	1.096	1.061~1.132
Region	Northeast vs. Northern	−0.440	0.029	228.171	<0.0001	0.644	0.608~0.682
	Eastern vs. Northern	−0.285	0.023	151.976	<0.0001	0.752	0.718~0.787
	Central vs. Northern	−0.312	0.028	128.830	<0.0001	0.732	0.694~0.772
	Southwest vs. Northern	−0.192	0.028	47.700	<0.0001	0.825	0.781~0.871
	Northwest vs. Northern	−0.252	0.027	84.594	<0.0001	0.777	0.737~0.820
	Southern vs. Northern	−0.547	0.034	262.321	<0.0001	0.579	0.541~0.618
Average annual household income	Moderate vs. Low	0.021	0.022	0.895	0.3442	1.021	0.978~1.066
	High vs. Low	0.046	0.022	4.552	0.0329	1.047	1.004~1.093
	Very high vs. Low	0.065	0.024	7.531	0.0061	1.067	1.019~1.118
BMI	Low vs. Normal	−1.841	0.263	−7.00	<0.0001	0.159	0.095~0.266
	Overweight vs. Normal	2.537	0.053	47.59	<0.0001	12.645	11.388~14.042
	Obese vs. Normal	5.198	0.085	60.86	<0.0001	180.989	153.025~214.064
Smoking	Former vs. Never	0.038	0.023	2.798	0.0944	1.038	0.994~1.085
	Current vs. Never	0.111	0.032	12.408	0.0004	1.117	1.05~1.188
Alcohol consumption	No current consumption vs. Current consumption	0.067	0.019	12.829	0.0003	1.069	1.031~1.109
Physical activity	Sufficient vs. Insufficient	0.199	0.024	71.565	<0.0001	0.819	0.782~0.858
Sleep duration	7~8 vs. ≤6	0.006	0.019	0.089	0.7650	1.006	0.968~1.045
	≥9 vs. ≤6	0.039	0.024	2.769	0.0961	1.040	0.993~1.089
Screen time	>5 vs. ≤5	0.084	0.025	11.696	0.0006	1.088	1.036~1.141

Abbreviations: BMI, body mass index.

## Data Availability

According to the regulations of the Institute of Nutrition and Health of the Chinese Center for Disease Control and Prevention, the data are not allowed to be disclosed.
